# Study on plugging law and plugging removal effect of pre filled screen in natural gas hydrate argillaceous silt reservoir

**DOI:** 10.1371/journal.pone.0344048

**Published:** 2026-06-04

**Authors:** Haoxian Shi, Jiudong Shi, Weigang Du, Fulong Ning, Wenwei Xie, Ranli Zhang, Yanjiang Yu

**Affiliations:** 1 Guangzhou Marine Geological Survey, Guangzhou, Guangdong, China; 2 National Engineering Research Center of Gas Hydrate Exploration and Development, Guangzhou, Guangdong, China; 3 China University of Geosciences, Beijing, China; 4 CNPC Offshore Engineering Company Limited, Beijing, China; 5 East China Petroleum Bureau of China Petroleum & Chemical Corporation, Nanjing, Jiangsu, China; NED University of Engineering and Technology, PAKISTAN

## Abstract

To address the issue of production decline caused by natural gas hydrate blockage in offshore screen pipes, optimization experiments for high-efficiency rotary water jet backflushing technology were conducted. The outer pressure and permeability of the screen pipes were used as criteria to assess backflushing effectiveness. The study examined the effects of various factors, including the type of backflush tool, tool movement speed, screen pipe type, and the presence of perforated pipes. The results revealed that, compared to high-pressure and low-pressure rotary spray guns, the high-pressure cavitation jet rotary flushing tool provided the best backflush performance. Backflushing effectiveness was higher at a tool movement speed of 1 m/min than at 2 m/min. Additionally, 30/40 mesh screen pipes demonstrated significantly better backflush performance than 40/70 mesh pipes. Screen pipes with perforated pipes showed lower backflush effectiveness compared to those without. These findings offer valuable insights for optimizing the backflushing process of offshore natural gas hydrate screen pipes.

## 1. Introduction

The hydrate reservoir in the Shenhu Sea area of the South China Sea is characterized by argillaceous silty sand with weak cementation and non-diagenetic features [1–3]. During hydrate extraction, the decomposition of hydrates near the wellbore leads to the migration of significant amounts of clay and silt. This migration can cause sand inflow, rendering the screen tube ineffective and resulting in wellbore blockage. Such blockages can interrupt extraction operations and reduce productivity. Therefore, selecting an appropriate method for plugging and removal is crucial to prevent sand control screen and wellbore blockage.

The rotating water jet blockage removal technology has utilized the jet dynamics generated by high-pressure water flow [4–7] to impact the screen tube or wellbore. This has been achieved through the high-speed water flow produced by the rotating nozzle, which has combined the effects of both high-pressure water and rotational motion to break and remove blockages. Previous research has extensively explored rotating water jet technology. For example, Zhanghongwei and colleagues have developed a high-pressure rotating water jet tool for removing blockages in sand control pipes. This tool has effectively flushed fine sand from the wellbore and has discharged blocked sand from the filling, thereby has cleared the reservoir and has restored productivity [8]. Zhu et al. [9] have designed a large-scale rotating water jet plug removal tool for a 244.5 mm wellbore, which has been capable of both hydraulic and pickling plug removal. The number of nozzles has been adjustable based on the specifications of the ground equipment. Weizenghong [10] has proposed a mobile rotary jet cleaning technology for oil pipes, enabling comprehensive cleaning of the inner wall by dragging a rotary nozzle through the pipe. Liululu et al. [11] have conducted a numerical simulation study on a self-propelled rotary jet nozzle. Their results have indicated that the diameter of the central hole in the rotary element has significantly affected drilling speed and efficiency. Shi [12] has proposed a technology that has combined hydraulic pulse and cavitation jet, which has effectively improved rock-breaking efficiency. Dong [13] designed a laboratory setup for evaluating the effectiveness of horizontal well de-blocking and investigated the qualitative relationship between de-blocking effectiveness and operational parameters such as the type of de-blocking fluid, injection volume, and displacement method. Shi [14] has analyzed the feasibility of applying composite plug removal technology in natural gas hydrate development, addressing the lack of research on the multi-component and multi-type plugging mechanisms in such contexts. Huang [15] has assessed the performance of controllable plasma pulse plug removal technology through indoor experiments that have simulated downhole screen plugging. This technology has effectively removed blockages, has reduced pressure behind the blockage, and has enhanced flow in the affected area. It has been suitable for various plugging conditions, including mild, moderate, and severe screen plate blockages. Tian et al. [16] have used a numerical model of the rotary jet field in the wellbore to investigate the impact of different parameters. Based on both simulation and experimental results, a rotary jet tool has been designed for dredging and descaling inefficient wells, and its field application has been carried out. The findings have indicated that rotary water jet technology has effectively removed blockages near wells and has enhanced permeability. This research has demonstrated that rotary water jet technology for plugging removal has been widely adopted, particularly in the cleaning of oil and gas wells. However, there has been limited research on the application of rotary water jets for the removal of blockages in natural gas hydrate wells, an area that has warranted further investigation. In this study, a plugging removal test using a rotary water jet on natural gas hydrate has been conducted. The effectiveness of the plugging removal has been primarily assessed based on the pressure outside the screen tube and the permeability of the screen tube. The impact of factors such as the type of plugging removal tool, tool movement speed, screen tube design, and perforated tube structure on the removal efficiency has also been explored.

## 2. Method

### 2.1 Blockage mechanism and characteristics

Blockages caused by natural gas hydrates during extraction arise from several factors, primarily due to the following reasons:

1) Composition of the Blockage: The blockage arises from the complex interplay of rock particles within the formation and the uncertain interaction of various fluid components. The formation remains stable until disturbed. However, when environmental parameters—such as temperature and pressure—alter, the formation can no longer maintain its original state. The ensuing interactions, including contact, chemical reactions, and aggregation among the components, initiate the blockage.
2) Repeated construction activities have disrupted the formation, leading to reactions between the original formation particles, fluids, and injected materials. These reactions result in blockages, including corrosion, microbial growth, and the accumulation of harmful inorganic ions. These substances gradually accumulate within formation pores or near the wellbore modification zone, where they are unable to be transported out by the fluid. As a result, they significantly reduce formation permeability.

During natural gas hydrate production, the resulting blockages exhibit the following characteristics:

1) The diversity and randomness of blockage types arise from the wide range of blocking substances, extensive affected areas, and multiple triggering factors, resulting in a certain degree of unpredictability. Of particular concern are nozzle blockages and the formation of “secondary hydrates” within 50 meters above and below the mudline in gas wells, as these can rapidly obstruct gas flow channels and disrupt production testing. The primary blockage mechanisms include: Scale blockage, Bacterial blockage, Emulsion blockage, Water blockage, Reservoir wetting reversal and Particle blockage [17].
2) The spatial distribution of blockage follows distinct patterns. Regardless of well type—whether vertical, horizontal, or other forms of production wells—larger particles and greater volumes of blocking material tend to accumulate near the production outlet during the spatiotemporal evolution of the blockage. When wellbore fluids are unable to transport sand, sand accumulation occurs. Human activities can also cause blockage, forming accumulation zones in the horizontal sections of the wellbore and near wellbore inclinations between 30° and 60°. Due to production effects, regions closer to the wellbore are more prone to blockage, while areas farther from the wellbore exhibit more stable formations, where pores are less likely to become blocked.

### 2.2 Theory of high-pressure rotating water jets

High-pressure water jets use water as a medium to generate a jet stream with high impact velocity and dynamic pressure, produced by specially designed nozzles. The jet exhibits significant pressure oscillations and rock-erosive effects, with vibration frequencies ranging from several thousand to tens of thousands of hertz. Pressure pulsation amplitudes can reach 24%–27%. Additionally, the cavitation effect in the jet creates instantaneous shock pressures that are 8.6 to 100 times higher than the impact pressure. When applied to wellbores, high-pressure rotating water jets dislodge blockages, expelling them with the fluid and effectively unblocking the well.

The nozzle, as the actuating component of a high-pressure water jet generator, facilitates energy conversion. By reducing the internal cross-sectional area, it concentrates the pressure energy of the high-pressure water flow, thereby increasing the kinetic energy of the ejected water jet. This results in a jet with superior flow characteristics and dynamic performance [18]. Under the operating conditions of high-pressure rotating water jets, the inlet pressure is much lower than the outlet pressure, the ratio of the nozzle outlet radius to the inlet radius is less than 1, and the water density is 998 kg/m^3^. The relationship between the velocity of the rotating water jet and the outlet pressure is expressed as follows [19]:


$$\begin{array}{c} v_{t}=\text{4}\text{4}.\text{7}\text{2}\sqrt{P} \end{array}$$ (1)

In the equation, vt represents the velocity of the rotating water jet, and P denotes the outlet pressure.

### 2.3 Plug removal simulation test

#### 2.3.1 Test device.

**Fig 1:**
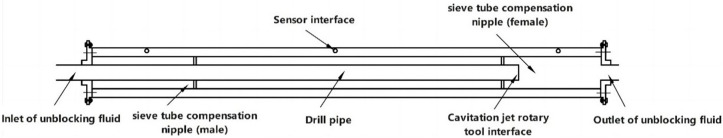
Schematic diagram of simulated wellbore.

**Fig 2:**
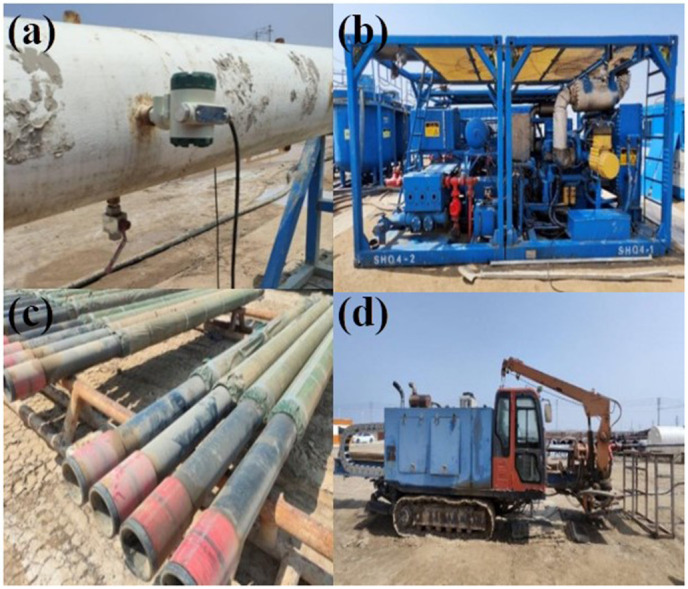
Test equipment. **(A)** AE-T pressure sensor **(B)** 600 pump injection pump **(C)** Trenchless drilling rig **(D)** sieves.

The simulation of the natural gas hydrate wellbore includes several components: a temperature and pressure monitoring system, a 600-liter injection pump, a traction device, a mixing tank, a liquid tank, a grit chamber, a trenchless drilling rig, and 30/40 mesh and 40/70 mesh screen pipes, along with three types of rotary jet tools. To ensure the reliability of the test results, the formation sand is derived from a mixture of in-situ sand and mud from the hydrate reservoir in the Shenhu Sea area of the South China Sea. The mass ratio of reservoir mud to water is 1:5, with a total of 750 kg of reservoir mud. A schematic diagram of the simulated wellbore is shown in Fig 1, while Fig 2 and Table 1 display the injection pump and trenchless drilling rig, and the test device parameters, respectively.

**Table 1:** Basic parameters of the unblocking test device.

Test device	Parameters	numerical value
Simulated wellbore	Size/m	7.3*0.2*0.2
	Sensor interface	M20*1.5
	Plug remover interface	M57
Sieves	Filling particles/mesh	40/60
	Effective percolation length/m	3.53
	Outer diameter/mm	114
	inside diameter/mm	88.9
Traction device	Moving speed/m/min	0-5
	Stroke/m	4

#### 2.3.2 Test principle.

**Fig 3:**
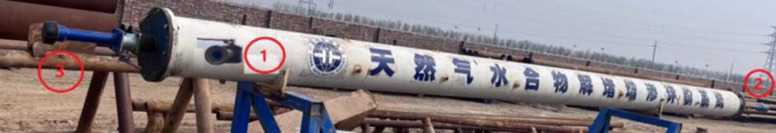
Schematic diagram of simulated wellbore for test.

The screen tube is positioned within the simulated wellbore, and the plugging agent, consisting of clean water mixed with reservoir mud and sand, is injected into the wellbore via an external pump for the purpose of plugging. Afterward, a plugging removal test is conducted. The simulated wellbore features three inlets and outlets, located at both ends and along the sides, as illustrated in Fig 3 Inlet 1 serves as the primary entry point for the plugging fluid, Inlet 2 connects to the traction device via a button-type 3 “1502 connector, and Outlet 3 serves as both the plugging fluid outlet and the fluid discharge point.

Calculate the pressure P1 after plugging and contamination of the test platform’s completion model, the pressure P2 after the first unblocking treatment, and the pressure P3 after the second unblocking treatment. During the test, record the pressure changes at these three points. Then, calculate the sieve tube permeability using Darcy’s law. The effectiveness of the unblocking treatments can be assessed by evaluating the outer pressure of the sieve tube during unblocking and plugging, as well as the changes in sieve tube permeability.The change in sieve tube permeability was as follows:


$$\begin{array}{c} {\text{k}}_{\text{si}}=\frac{{\text{q}}_{\text{i}}\mu }{\text{2}\pi h\Delta {\text{p}}_{\text{i}}}\text{ln}\frac{{\text{r}}_{\text{0}}}{{\text{r}}_{\text{i}}} \end{array}$$ (2)

In the formula: μ—viscosity of the test fluid, Pa·s; h—the effective percolation length of the short section of the test sieve tube, m; ${\text{r}}_{\text{0}}$—the outer radius of the short section of the test sieve tube, m; ${\text{r}}_{\text{i}}$—the inner radius of the short section of the test sieve tube, m; ${\text{q}}_{\text{i}}$—flow rate through the test sieve at the ith instant, $m^{\text{3}}$/s; $\Delta {\text{p}}_{\text{i}}$—average of the difference of the pressure P_1_, P_2_ and P_3_, Pa; ${\text{k}}_{\text{si}}$—permeability of the short section of the test sieve at the ith instant, $m^{\text{2}}$.

### 2.4 Test flow

#### 2.4.1 Water injection.

The test bore is initially subjected to a pressure of 5 MPa and stabilized for 5 minutes. The screen pipe is then loaded onto the test platform. After loading, a pressure transducer is placed at the inlet end of the test apparatus (positions 1–7) to measure the pressure. Fresh water is injected into the screen tube annulus at position 1 (2“1502). Once the pressure stabilizes at all seven positions, the flow rate Q is measured. The initial permeability of the simulated layers at each segment is then calculated using Darcy’s law and is recorded as K_1_.

#### 2.4.2 Plugging test.

**Fig 4:**
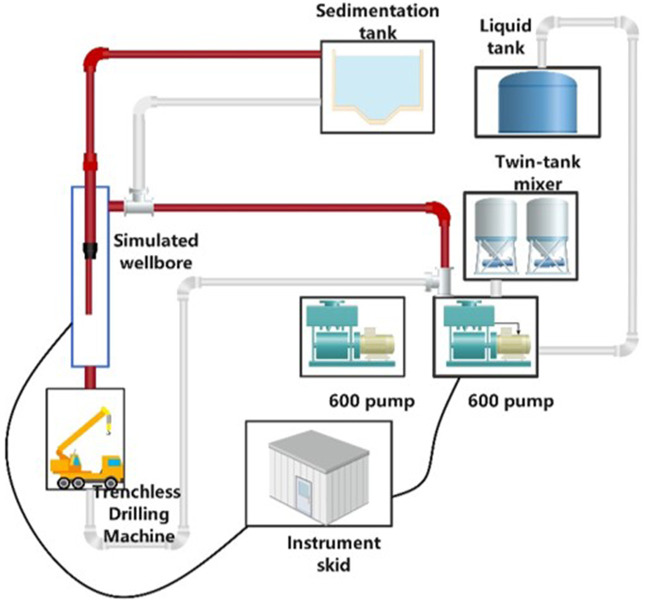
Plugging process diagram.

The screen pipe is plugged by injecting a mixed plugging liquid, prepared by continuously stirring a 1:5 ratio of plugging agent to water to ensure uniformity. The pressure is measured at each point, and the plugging process continues until the pumping pressure reaches 6 MPa. At this point, the displacement is recorded, and the plugging process is stopped. The corresponding pressure values are documented in the table below. Using Darcy’s law, the permeability (K_2_) for each section of plugging is then calculated. A schematic diagram of the plugging process is presented in Fig 4.

Before proceeding with the decongestion, repeat the plugging test once the pressure and permeability values have stabilized and are consistent with the initial measurements. Collect all relevant data, record any significant changes in real-time, and calculate the permeability (K_3_) for each section after decongestion. The test should be terminated when no further changes in permeability (K_3_) are observed.

#### 2.4.3 Unblocking test.

**Fig 5:**
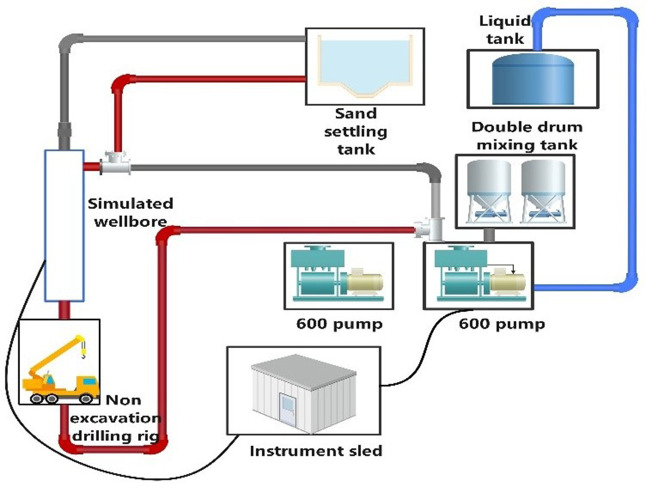
Schematic diagram of plug removal process.

The congestion relief flowchart is shown in Fig 5, The specific steps are as follows.

(1) Debug the pump injection and dragging equipment: Configure the unblocking liquid, establish the injection process for the blocking liquid, and simulate the construction dragging procedure.
(2) Simulate on-site construction with a non-excavation drilling rig: Operate the drilling rod at a constant speed, alternating its direction to drive the center rod back and forth, completing one full cycle of reciprocation.
(3) Initiate unblocking with water: During the unblocking stage, collect a sample at the exit of the screen pipe test stand after one reciprocation. Monitor the pressure changes at three test points and record all data. Document significant data fluctuations on-site, and calculate the permeability K_3_ for each section after unblocking.
(4) Analyze the unblocking effect: If no significant pressure change is observed after three reciprocations, terminate the unblocking test. Record the pressure changes at the three test points, document key data fluctuations, and calculate the permeability K_3_ for each section after unblocking.
(5) Complete the test: Once there is no further change in permeability K_3_, stop the test. Install a plug, switch to the next tool, and repeat the above steps.

### 2.5 Equipment testing

The testing equipment underwent multiple validation tests and demonstrated high reliability. Before each experiment, the equipment was calibrated to ensure measurement accuracy. During testing, a 40/70 mesh screen tube was deliberately clogged under a flow rate of 120 L/min until the external pressure reached 6 MPa. The cleaning process was performed using Tool 3 (High-Pressure Cavitation Jet Rotating Flushing Tool) equipped with seven 6 mm nozzles and operated at a travel speed of 1 m/min. Pressure variations were recorded, and permeability rates were calculated at different stages. The error margins across three test runs were minimal, indicating good repeatability.

The test results are presented in Table 2, Based on the pressure variations inside and outside the screen after unblocking, the post-unblocking pressures in the three tests decreased by 98.32%, 96.22%, and 94.34% relative to the blocked conditions. The corresponding permeabilities of the screen after unblocking were 191.2 mD, 194.5 mD, and 190.2 mD, respectively. In all three tests, the pressure difference across the screen decreased by more than 90% compared with the blocked state, indicating a substantial restoration of flow capacity. The permeability of the screen increased significantly after unblocking, with pressure measurement errors remaining within 5%. Under identical operating conditions, the differences in permeability among the three tests were negligible.

**Table 2:** Testing and trials.

Number of tests	Plugging pressure P1 (MPa)	Plugging permeability K_2_ (mD)	Pressure after plugging P2 (MPa)	Permeability after decongestion K_3_ (mD)	(P1-P2)/P1 × 100%
1	5.94	3.86	0.1	191.2	98.32%
2	6.10	3.69	0.23	194.5	96.22%
3	6.01	3.71	0.34	190.2	94.34%

## 3. Test results and analysis

### 3.1 Types of unblocking tools

The operational principles of different tools [20] are shown in Table 3.

**Table 3:** Introduction to plugging removal tools.

	Tool Name	operational principle
Unblocking tool 1	High-pressure rotary spray gun	The internal rotation is driven by a gear reduction mechanism to rotate the ball head to achieve a predetermined spraying angle by spreading the spray nozzles evenly over the ball head at 120°.
Unblocking tool 2	Low-pressure rotary gun	After fluid is fed into the mounting base, the rotating ball head is driven by the torque generated by the unevenly distributed nozzles, thus realizing the rotation of the nozzles.
Unblocking tool 3	High-pressure cavitation jet rotary flushing tool [21]	Combines high-pressure water flow with cavitation effect and rotary motion to generate a powerful impact force through high-pressure water flow.

**Fig 6:**
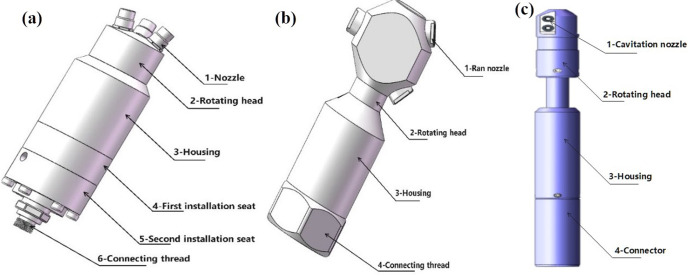
Structure diagram of three plugging removal tools. **(A)** High-pressure rotary spray gun **(B)** Low-pressure rotary gun **(C)** High-pressure cavitation jet rotary flushing tool.

The structure of the three tools is shown in Fig 6.

**Fig 7:**
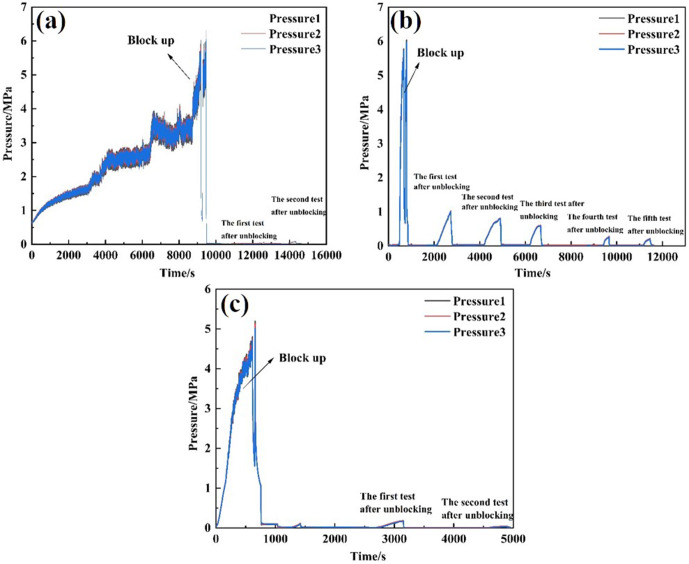
Pressure curve outside the screen during plugging and plugging removal by different plugging removal tools. **(A)** High-pressure rotary spray gun **(B)** Low-pressure rotary gun **(C)** High-pressure cavitation jet rotary flushing tool.

**Fig 8:**
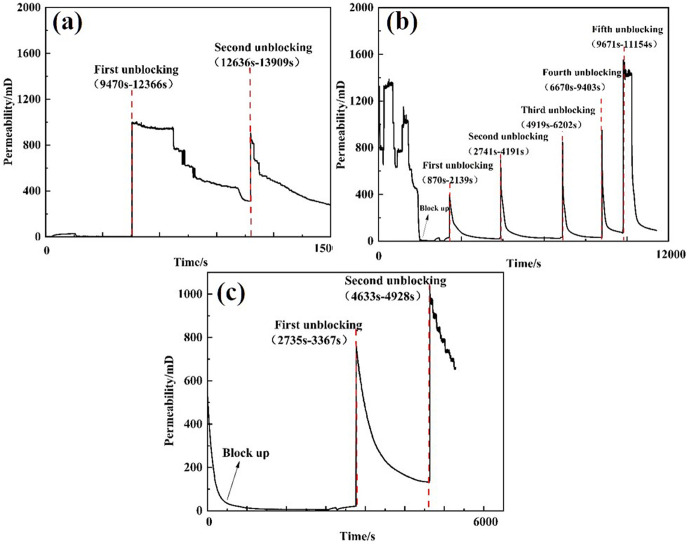
Permeability curve of plugging and plugging removal screens by different plugging removal tools. **(A)** High-pressure rotary spray gun **(B)** Low-pressure rotary gun **(C)** High-pressure cavitation jet rotary flushing tool.

Three types of unblocking tools were employed to evaluate the unblocking effectiveness of clear water plugging liquid. Pressure changes were recorded, and the permeability after unblocking was calculated. The test results are presented in Table 4. The external pressure of the screen pipe during the blocking and unblocking process is illustrated in Fig 7, while the permeability of the screen pipe is shown in Fig 8.

**Table 4:** Plugging process data of three tools.

Tool type	Plugging pressure P_1_ (MPa)	Plugging permeability K_2_ (mD)	Pressure after plugging P_2_ (MPa)	Permeability after decongestion K_3_ (mD)	(P1-P2)/P1 × 100%
High-pressure rotary spray gun	6.13	3.74	0.09	254.93	98.53%
Low-pressure rotary gun	5.98	3.84	0.2	114.72	96.66%
High-pressure cavitation jet rotary flushing tool	5.07	4.53	0.04	478	99.21%

From the perspective of pressure changes on the inner and outer sides of the screen tube after decongestion, the pressure of the three decongestion tools decreased by 98.53%, 96.66%, and 99.21%, respectively, relative to the initial plugging pressure. Regarding the permeability of the screen tube post-decongestion, the permeability values for the three tools were 254.93 mD, 114.72 mD, and 478 mD [22], respectively. The decongestion effect of the high-pressure rotary spray gun was relatively stable, as it employed high-pressure water jet impact for blockage removal. The low-pressure rotary gun, however, performed less effectively due to its insufficient pressure, which resulted in failure to rotate and, consequently, a lower decongestion efficiency compared to the other two tools. In contrast, the high-pressure cavitation jet rotary flushing tool provided the most complete blockage removal, as it combined high-pressure water flow with cavitation, resulting in superior unblocking performance.

### 3.2 Different moving speed of tools

In horizontal well plugging operations, the tool typically moves back and forth at a constant speed. The injector speed is usually set between 1 and 2 m/min, with 3–4 round trips performed. This process is used to analyze permeability changes at different movement speeds.

**Fig 9:**
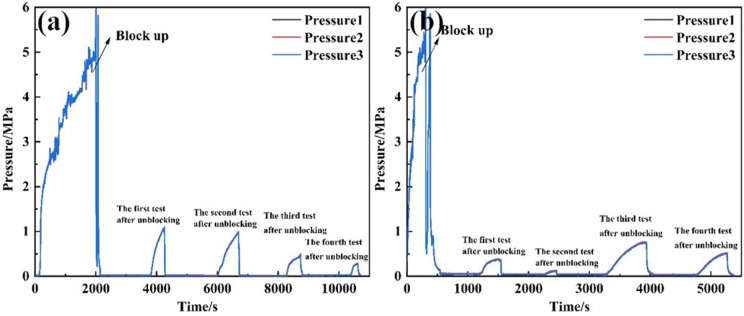
Pressure curve outside the screen tube during plugging and removing process of tool 3 at different moving speeds. **(A)** 1m/min **(B)** 2m/min.

**Fig 10:**
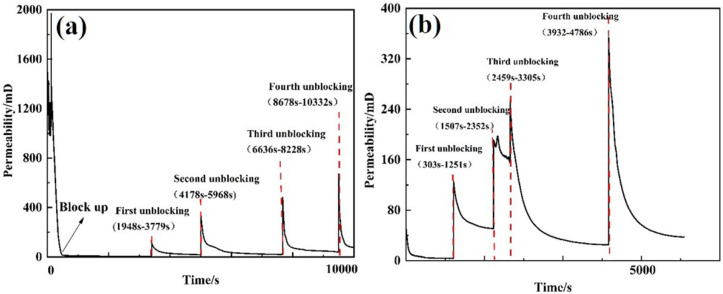
Permeability curve of sieve tube during plugging and plugging removal of tool 3 at different moving speeds. **(A)** 1m/min **(B)** 2m/min.

Under a flow rate of 120 L/min, the 40/70 mesh screen pipe was subjected to plugging, reaching a pressure of 6 MPa, as measured outside the screen pipe. The plugging test was performed using Tool 3 (a high-pressure cavitation jet rotary flushing tool) with a 6 mm*7 nozzle. Pressure changes were recorded throughout the process, and permeability was calculated at various stages. The test results are presented in Table 5. The pressure variations outside the screen pipe during both the plugging and unplugging processes are shown in Fig 9, while the permeability of the screen pipe is illustrated in Fig 10.

**Table 5:** Plugging process data of tool 3 at different moving speeds.

Tool speed (m/min)	Plugging pressure P1 (MPa)	Plugging permeability K_2_ (mD)	Pressure after plugging P2 (MPa)	Permeability after decongestion K_3_ (mD)	(P1-P2)/P1 × 100%
1	5.94	3.86	0.1	191.2	98.32%
2	6.17	3.72	0.51	37.49	91.73%

**Fig 11:**
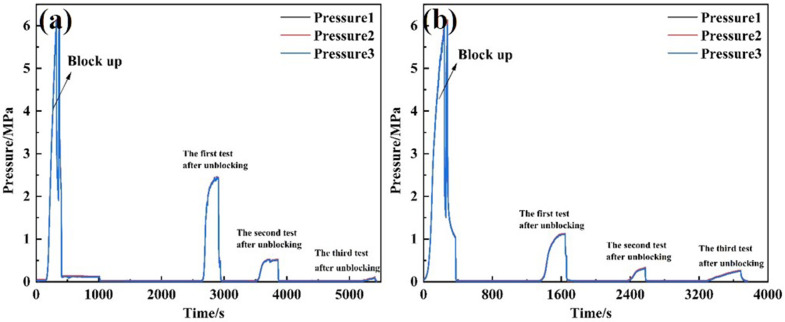
Pressure curve outside the screen tube in the process of plugging and removing with different moving speeds of tool 1. **(A)** 1m/min **(B)** 2m/min.

**Fig 12:**
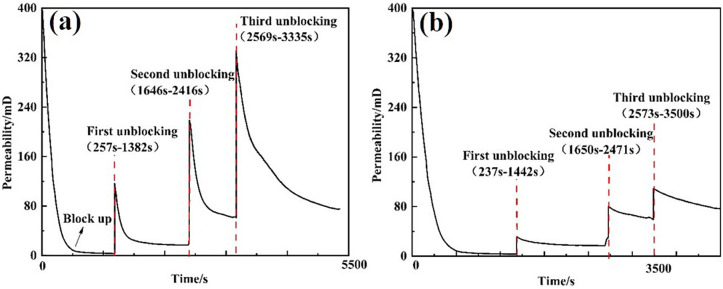
Screen permeability curve of plugging and removing process of tool 1 at different moving speeds. **(A)** 1m/min **(B)** 2m/min.

Under a displacement rate of 120 L/min, the 30/40 mesh screen pipe (equipped with a perforated pipe) was obstructed until the external pressure reached 6 MPa. Unblocking tests were then conducted at speeds of 1 m/min and 2 m/min using Tool 1 (a high-pressure rotary spray gun with three 6 mm nozzles). Pressure changes were recorded, and permeability was calculated at different stages. The test results are presented in Table 6. The pressure variation on the exterior of the screen pipe during both clogging and unblocking is illustrated in Fig 11, while the permeability of the screen pipe is shown in Fig 12.

**Table 6:** Plugging process data of tool 1 at different moving speeds.

Tool speed (m/min)	Plugging pressure P1 (MPa)	Plugging permeability K_2_ (mD)	Pressure after plugging P2 (MPa)	Permeability after decongestion K_3_ (mD)	(P1-P2)/P1 × 100%
1	6.17	3.72	0.1	191.2	98.38%
2	6.08	3.77	0.26	73.54	95.72%

After unblocking the screen tube, the pressure difference between the inside and outside decreased by 98.32% relative to the blocking pressure. For Tool 3, the permeability of the screen tube was 191.2 mD at a moving speed of 1 m/min, representing a 91.73% reduction relative to the blocking pressure. For Tool 1, at a speed of 2 m/min, the permeability decreased to 37.49 mD, a 91.73% reduction compared to the blocked state.

At a moving speed of 1 m/min, the post-blocking pressure decreased by 98.38% relative to the blocking pressure, with the permeability of the screen tube measuring 191.2 mD. At a speed of 2 m/min, the post-blocking pressure dropped by 95.72%, and the permeability decreased to 73.54 mD.

Tests conducted using two tools on two different screen tubes showed that blocking was more effective at a speed of 1 m/min than at 2 m/min. This is because the slower speed allows the tool to remain in contact with the plugged material longer, thereby increasing the flushing action on the material outside the screen tube.

### 3.3 Screen tube types

**Fig 13:**
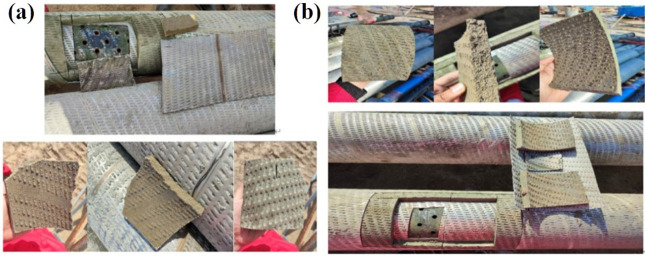
Comparison of cutting appearance of different sieves. **(A)** 30/40 mesh sieve **(B)** 40/70 mesh siev.

“30/40 mesh and 40/70 mesh screen tubing are two common sizes used in oil and gas drilling to control the size of sand particles in wellbore fluids, preventing wall damage and protecting equipment. The 30/40 mesh screen tubing allows particles of up to 40 mesh, with an approximate pore size of 0.425 mm, to pass through. In contrast, the 40/70 mesh screen tubing permits particles of up to 70 mesh, with an approximate pore size of 0.212 mm, to pass. The construction of the screen tube is illustrated in Fig 13.

**Fig 14:**
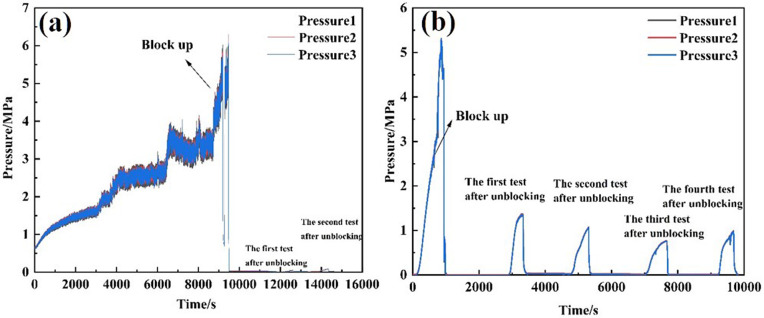
Pressure curve outside the screen tube during plugging and unplugging of different screens. **(A)** 30/40 mesh sieve **(B)** 40/70 mesh sieve.

**Fig 15:**
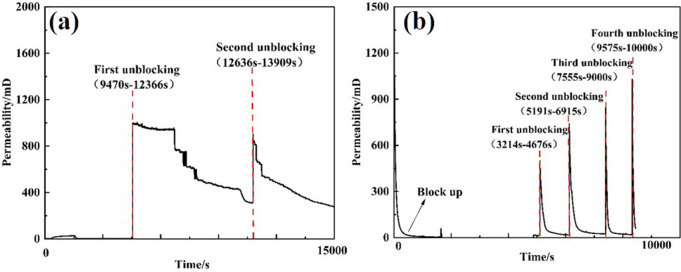
Screen permeability curve of different screen plugging and plugging removal processes. **(A)** 30/40 mesh sieve **(B)** 40/70 mesh sieve.

The plugging of 30/40 mesh and 40/70 mesh screen tubes was tested under a discharge rate of 120 L/min, with an external pressure of 6 MPa applied to the screen tube. To assess unblocking performance, Tool 1 (equipped with three 6-mm nozzles) was used at 100 L/min. During the test, pressure changes were recorded, and permeability was calculated at various stages. The results are summarized in Table 7. The pressure variations outside the screen tube during both clogging and unblocking processes are illustrated in Fig 14, while Fig 15 shows the corresponding permeability values.

**Table 7:** Plugging process data of different sieves.

Sieve tube (m/min)	Plugging pressure P_1_ (MPa)	Plugging permeability K_2_ (mD)	Pressure after plugging P_2_ (MPa)	Permeability after decongestion K_3_ (mD)	(P1-P2)/P1 × 100%
30/40 mesh	6.13	3.74	0.09	254.93	98.53%
40/70 mesh	5.27	4.53	0.98	23.41	81.40%

From the perspective of pressure changes on the inner and outer sides of the screen tube after unblocking, the pressure of the 30/40 mesh screen tube decreased by 98.53% compared to its blocked state, and its permeability increased to 254.93 mD. In contrast, the pressure of the 40/70 mesh screen tube after unblocking decreased by 81.40% relative to the blocked pressure, with a permeability of 23.41 mD. The 30/40 mesh screen demonstrated a more significant improvement in performance after unblocking due to its larger pore size, which allows larger particles to pass through more easily.

### 3.4 Whether to install perforated pipe

Perforated tubing is commonly used in oil and gas fields to enhance fluid flow by providing additional channels for fluid passage, thereby reducing or preventing clogging caused by low flow rates. However, its effectiveness may vary, as perforated tubing is not universally applicable and can be less efficient in some cases due to poor design or other factors. To assess the impact of perforated tubing on unblocking performance, a comparison was made between 30/40 mesh screen tubes with and without perforations.

**Fig 16:**
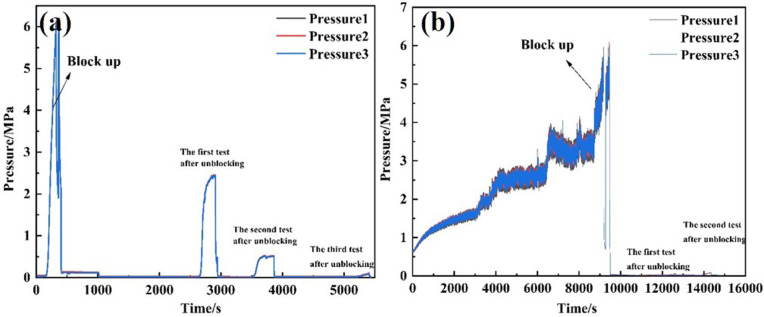
Pressure curve outside the screen tube during plugging and plugging removal with or without perforated pipe. **(A)** 30/40 mesh (Perforated pipe installed) **(B)** 30/40 mesh (No perforated pipe installed).

**Fig 17:**
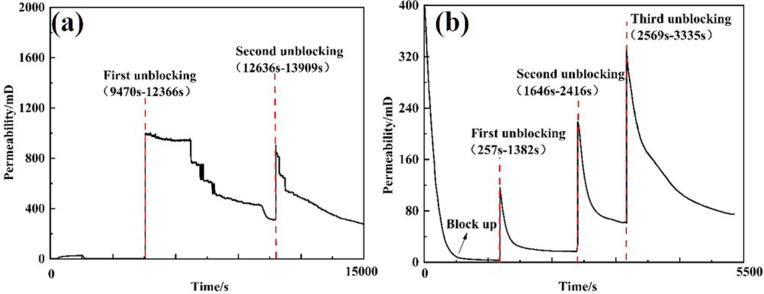
Pressure curve of outside screen tube during plugging and plugging removal with or without perforated pipe. **(A)** 30/40 mesh(Perforated pipe installed) **(B)** 30/40 mesh(No perforated pipe installed).

The clogging tests of 30/40 mesh screens—both with and without a perforated tube—were conducted under a displacement rate of 120 L/min, until the external pressure on the screen reached 6 MPa. Subsequently, the clogging test was performed at 100 L/min using Tool 1 (6 mm nozzle × 3). Pressure changes were recorded at different stages, and permeability was calculated. The results are presented in Table 8. Fig 16 shows the external pressure variations during the plugging and unplugging processes, while Fig 17 illustrates the permeability of the screen tube.

**Table 8:** Process data of plugging removal with or without perforated pipe.

Sieve tube (m/min)	Plugging pressure P1 (MPa)	Plugging permeability K_2_ (mD)	Pressure after plugging P2 (MPa)	Permeability after decongestion K_3_ (mD)	(P1-P2)/P1 × 100%
30/40 mesh (Perforated pipe installed)	6.17	3.72	0.1	191.2	98.38%
30/40 mesh (No perforated pipe installed)	6.13	3.74	0.09	254.93	98.53%

From the perspective of pressure changes on the inner and outer sides of the screen tube after unblocking, the pressure of the 30/40 mesh screen tube decreased by 98.38% following the installation of the perforated pipe, relative to the blocked pressure. The permeability of the screen tube after unblocking was 191.20 mD [23]. For the 30/40 mesh screen tube, the pressure decreased by 98.53% after unblocking, with a permeability of 254.93 mD. The unblocking effect after installing the perforated pipe was less than that observed without the perforated pipe. This reduced effectiveness is attributed to the tool nozzle’s unblocking action, which only occurs when it directly faces the perforated tube’s eye. This limited positioning shortens the flushing time, resulting in a decrease in the overall unblocking effect.

### 3.5 Analysis of factors affecting the effect of unblocking

Synthesize the results of data analysis at each test stage and provide recommendations regarding the unblocking tool, its movement speed, screen tube selection, and the necessity of installing a perforated tube.

#### 3.5.1 Influence of the type of unblocking tool.

Under identical conditions, the high-pressure cavitation jet rotary flushing tool (Tool 3) outperforms Tool 1(high-pressure rotary gun)and Tool 2 (low-pressure rotary gun) in terms of unblocking effectiveness. Among all the tools, Tool 3 demonstrates the best unblocking performance. The combination of high-pressure water flow, cavitation, and rotary motion proves particularly effective for removing harder sediments, dirt, or long-term blockages. All three tools employ rotational motion to generate spiral jet trajectories, producing a substantially wider dispersion angle than that of fixed jets. However, Tool 2 (low-pressure rotary gun) exhibits rapid energy decay, restricting its effectiveness to the shallow surface layer of the blockage. In contrast, Tool 1 (high-pressure rotary gun) and Tool 3 (high-pressure rotary spray gun) display slower jet energy decay and longer effective ranges, allowing penetration through surface pores and energy delivery to deeper blockage layers. Moreover, Tool 3 achieves “deep-layer disruption capability” because its jet contains cavitation bubbles. These bubbles move with the flow field into high-pressure regions, where they collapse and release instantaneous energy. The superior performance of Tool 3 arises not from a single mechanism but from the synergy between jet dynamics and cavitation: jet dynamics provide the energy base and transmission pathway for cavitation, while cavitation, in turn, enhances jet dynamics by promoting deep-layer disruption. Together, these coupled effects constitute the unblocking process.

#### 3.5.2 Effect of tool movement speed.

The plugging effect of the 30/40 mesh screen tube (perforated tube) was better when using Tool 1 (high-pressure rotary gun) with a 6 mm nozzle (×3) at a speed of 1 m/min, compared to a speed of 2 m/min. Similarly, for the 40/70 mesh screen tube, Tool 3 (high-pressure cavitation jet rotary rinsing tool) with a 5 mm nozzle (×7) showed better plugging performance at a speed of 1 m/min than at 2 m/min.

A lower tool speed (1 m/min) provided superior unblocking results compared to higher speeds (2 m/min). This reduced speed increases the contact time between the tool and the obstruction, allowing for more thorough removal. In cases of hard or complex blockages, the extended fluid exposure time enhances the unblocking and loosening effects. Additionally, traveling at a uniform speed of 1 m/min results in smoother operation and reduces the impact on equipment, helping to prolong the lifespan of both unblocking equipment and downhole tools.The tool’s movement speed directly influences the duration of its action per unit area on the clogged section of the screen pipe. A lower speed increases both the energy input and the duration of physicochemical reactions. In contrast, at higher speeds (e.g., 2 m/min), the tool is more susceptible to minor vibrations and trajectory deviations caused by inertia. These effects reduce the overlap of high-pressure jet scans across the screen surface, leading to localized areas that remain untreated. Conversely, a lower movement speed (1 m/min) promotes smoother tool motion and enables the jets to generate continuous, uniform, spiral-shaped trajectories along the screen surface. This ensures adequate energy input and fluid contact for each pore.

#### 3.5.3 Screen tube effectiveness.

The 30/40 mesh screen is more effective than the 40/70 mesh screen when using Tool 1. The 30/40 mesh screen features a relatively larger aperture (typically between 0.42 and 0.50 mm), allowing larger clogging particles to pass through while effectively removing larger silt and sand particles. In contrast, the 40/70 mesh screen has a significantly smaller aperture (typically between 0.25 and 0.42 mm), which is designed to handle finer particles. However, due to its smaller pore size, it is more prone to clogging by larger particles, resulting in reduced efficiency in particle removal. Tool 1 fragments large blockages into smaller particles during the unblocking process, resulting in a broader particle size distribution. The aperture size of the screen pipe directly determines whether these fragmented particles cause secondary blockages, thereby influencing the stability of the final unblocking outcome. A larger aperture improves blockage removal efficiency by preventing particle retention, reducing deep-seated blockages to facilitate effective energy transfer, and minimizing the risk of secondary blockages, thus ensuring stable and reliable unblocking performance.

#### 3.5.4 Effect of perforated pipe.

The installation of the perforated pipe reduces the effectiveness of unclogging. It may also interfere with the structure of the screen tube. The tool nozzle is effective in unclogging the screen tube only when positioned directly in front of the perforated pipe’s eyelet. Additionally, the perforated pipe may cause clogging particles to accumulate near the eyelet. The primary function of the unblocking tool depends on high-pressure jets that transfer energy to the blockage. However, the perforated pipe acts as a barrier to jet propagation, resulting in energy attenuation and disturbance before the jets reach the screen pipe. Consequently, the effective energy density applied to the blockage is reduced. The solid wall sections of the perforated pipe obstruct the jet stream from the unblocking tool, leaving parts of the blocked screen pipe completely unexposed to the jets. Meanwhile, jets striking the perforated wall are reflected and scatter irregularly, deviating from their intended trajectory. These reflections not only fail to reach the blocked area but may also interfere with subsequent jets, further decreasing the overall jetting efficiency.

## 4. Conclusion

To address wellbore blockage caused by the natural gas hydrate extraction process, a rotary water jet high-efficiency decongestion test was conducted. The study examined the effects of various factors, including the type of decongestion tool, tool movement speed, screen pipe type, and the presence or absence of a perforated pipe, on the decongestion performance of the screen pipe. The key findings are as follows:

(1) Effectiveness of the High-Pressure Cavitation Jet Rotary Flushing Tool:Under identical conditions, the pressure difference between the inside and outside of the screen pipe decreases by 98.53% after using the high-pressure cavitation jet rotary flushing tool, which demonstrates the best decongestion performance compared to both the high-pressure rotary gun and the low-pressure rotary gun.
(2) Influence of Tool Speed on Permeability:The moving speed of the plugging tools significantly affects the permeability within the screen tube. When Tool 1 and Tool 3 move at 1 m/min, the differential pressure inside and outside the screen tube decreases by 98.38% and 95.12%, respectively. At a speed of 2 m/min, the differential pressure decreases by 91.73% and 95.72%, respectively, indicating a better unblocking effect at the lower speed.
(3) Impact of Screen Tube Type on Permeability:The type of screen tube notably influences the permeability after unblocking. Under the same conditions, the internal and external pressure differential of a 30/40 mesh screen tube decreases by 98.53%, while the pressure differential for a 40/70 mesh screen tube decreases by only 81.40%. Thus, the unblocking effect is more effective in the 30/40 mesh screen tube.
(4) Effect of Perforated Tube on Permeability:The presence of a perforated tube slightly influences the permeability condition within the screen tube after unblocking. Under identical conditions, the pressure difference inside and outside the screen tube is marginally higher when no perforated tube is used, suggesting that unblocking is more effective without the perforated tube.
