# Correction: Study on plugging law and plugging removal effect of pre filled screen in natural gas hydrate argillaceous silt reservoir

**DOI:** 10.1371/journal.pone.0354268

**Published:** 2026-07-20

**Authors:** Haoxian Shi, Jiudong Shi, Weigang Du, Fulong Ning, Wenwei Xie, Ranli Zhang, Yanjiang Yu

There is an error in affiliation 3 for authors Haoxian Shi, Jiudong Shi, and Fulong Ning. The correct affiliation 3 is: China University of Geosciences, Wuhan, China.

The Funding statement for this article is incorrect. The correct Funding statement is as follows: This research was financially sponsored by the National Natural Science Foundation of China (Grant Nos. U2444216), Major Basic and Applied Basic Projects of the Guangdong Provincial Department of Science and Technology (Grant Nos. 2025MGS-HBZ-001), Science and Technology Projects in Key Areas of Nansha District (Grant Nos. 2023ZD017), and the Geological Survey Projects of China Geological Survey (Grant Nos. DD20221700 and DD20230063).
